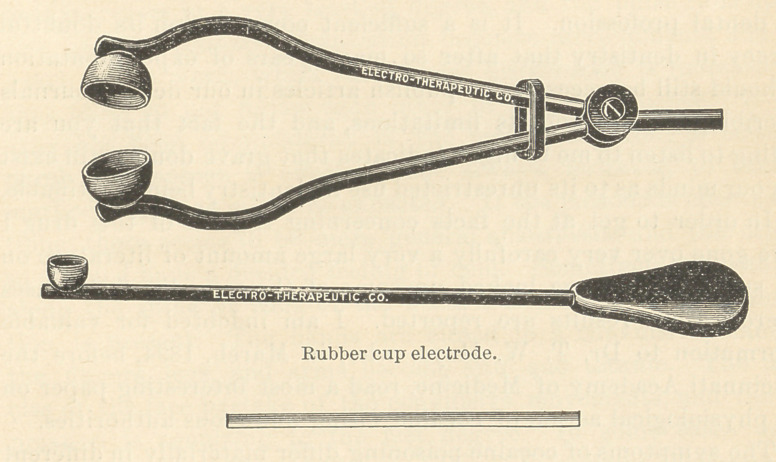# The Principles and Applications of Cataphoresis

**Published:** 1896-07

**Authors:** Wendell C. Phillips

**Affiliations:** New York


### THE PRINCIPLES AND APPLICATIONS OF CATAPHORESIS.[1]

BY WENDELL C. PHILLIPS, M.D., NEW YORK.[2]

The subject of cataphoresis, or the cataphoric action of electricity, is by no means new. It was known and used to some extent before the present generation was born. The use of the electrical current in anæsthesia was known as far back as 1859. If any of you care to look up the matter, you may refer to articles in the *Medical Times* for February 12 and June 25, 1859, by Dr. Richardson. You may learn something of the anæsthetic effect of the continuous current as used by him; not, however, with the use of the remedies that we use at this time, but with aconite and chloroform. It is recorded that he performed, among other things, one or two extractions, and it is reported that the operations were done without pain. I would also refer you to an interesting article by Dr. Frederick W. Peterson in Bigelow's "International System of Electro-Therapeutics," which is the latest book on the general subject of electricity that we have. Later on we have most admirable articles by Dr. Henry W. Gillett,[3] of Newport, upon the subject of obtunding sensitive dentine. Then you will find much useful information upon the general subject in the paper recently published

---

[1] Read before the Central Dental Association of Northern New Jersey, March 16, 1896.

[2] Lecturer upon Diseases of the Nose and Throat, New York Post-Graduate Medical School and Hospital; Assistant Surgeon to the Manhattan Eye and Ear Hospital.

[3] Dental Cosmos, February, 1896; INTERNATIONAL DENTAL JOURNAL, February, 1896.

in the *Dental Cosmos*,[1] by Dr. William J. Morton, who has presented the matter in his usual intelligent and accurate manner. Allow me to give you three definitions of cataphoresis. That of Dr. Peterson is as follows: "Cataphoresis is the introduction of medicaments by means of electricity into the body through the skin or mucous membrane." In the light of recent developments his definition is not complete, for we have established the fact that medicaments can be introduced into the hard tissues by means of electricity. Dr. Morton's definition is: "The movement of fluids, together with the substances they may hold in solution, from the positive pole of electrodes conveying a continuous current in tissue towards the negative pole." Dr. Gillett modifies these two by including the teeth. It seems to me it might well be described as the introduction into both the hard and the soft tissues, by means of a continuous current, of fluid medicaments from the positive pole without the breaking up of their constituent elements.

You will naturally have to consider, in the study of cataphoresis, two other properties: one is osmosis, the other electrolysis. Osmosis may very well be demonstrated by a simple illustration, which is by no means new. We will suppose that you have a jar of fluid something like this (drawing on black-board). Into this jar of fluid you place a porous diaphragm, which divides it into two compartments. You put into one compartment one kind of fluid, and into the other compartment another kind, the two fluids having different densities. The lighter fluid will pass through the porous diaphragm to the fluid of greater density, as may be easily seen if the two fluids are of different colors. This passing of a fluid through a porous diaphragm, or similar intervening medium, to unite with another fluid, is what is called osmosis. Now, if you drop into the lighter of the two fluids the positive pole of a continuous current of electricity, and into the other fluid the negative pole of the continous current, and turn on the current, you will find that the osmotic action will go on with much greater rapidity than before; the electricity hastens the osmotic action of the fluids. That is cataphoresis. Cataphoresis is electrical osmosis, or osmosis hastened by the action of the continuous current. Not only that, but having two fluids, one denser than the other, you may, by reversing the poles of the battery, overcome this natural osmotic action and cause the denser fluid to pass towards the lighter, instead of the lighter flowing towards the denser. Therefore, you may keep in your minds

---

[1] Dental Cosmos, January, 1896.

this simple fact, that cataphoresis is nothing more than electrical osmosis, and it is purely a mechanical process.

Electrolysis and cataphoresis are both brought about by the same kind of current under somewhat similar conditions. But you have to bear this in mind, that where you attempt electrolysis you use different remedies and different electrodes. Electrolysis means the electrical decomposition of chemical compounds. If you take an oxidizable metal, like copper or zinc, for the positive pole, and put it in contact with living tissue, there will be oxidization of the copper, which will remain in contact with the tissues. In cataphoresis you use an electrode that is not readily oxidized. Platinum or gold should be used in the cataphoric introduction of medicaments.

The question will come up, Why do we use a continuous current? Continuous current and galvanic current mean the same thing, but the term continuous current is preferable. It is better than the term galvanic current, for the reason that you do not use it for galvanism all the time. There are many illustrations that might be used to demonstrate the difference between cataphoresis and ordinary osmosis. One is to take two pieces of blotting-paper, putting some preparation like pyrozone on one and staining the other with permanganate of potassium; place the two pieces end to end and observe the osmotic effect: the pyrozone will pass into the end of the permanganate of potassium piece of paper for a line or two, illustrating the osmotic action. Now, if you apply the positive electrode to the pyrozone side and the negative pole to the permanganate side, a rapid decolorization will take place, showing that the current is driving the lighter towards the denser fluid, and much further than would be done by ordinary osmosis.

Again, if you place the positive pole, which we may speak of as the dry pole, to one end of a hard-boiled egg, and the negative pole to the other end of the egg, you will see the fluids collect at the negative pole. One of the most recent theories that I may call to your minds to explain the cataphoric process is that there must be a movement of fluids from the positive towards the negative pole, and that, as the fluid moves, there are little vacuums formed, and the fluid medicament rushes in to fill these vacuums. I do not know whether it is true or not.

I am frequently asked if it is possible to have both electrolysis and cataphoresis simultaneously. In all cases of cataphoresis you do get some electrolytic effect at the same time. Electrolysis and cataphoresis are almost always combined; but when cataphoric electrodes are used, the electrolytic effect is so slight that it is not perceptible. An illustration of this combined action may be seen if you place a copper or a zinc electrode in contact with a piece of meat. There will soon be a deposit of oxides in the meat and at the same time there will also be some movement of these oxides into the mass of meat. Now, as far as the oxidization of the electrode is concerned, that is electrolysis, but the moment you get any movement, as the result of the breaking down of this metal, towards the negative pole, that movement is cataphoresis.

In cataphoresis, the quantity of the medicament introduced will depend both upon the strength of the current used and upon the density of the tissue. The less the density of the tissue the more of your medicament you will be able to introduce, and the stronger the current the more medicament you will be able to introduce.

In order to fully understand the measurements of the current we use the words volt, ampère, and ohm. For a long time they were to me great bugbears. I will try to explain them to you. The voltage of a current means its pressure, and by ampères we measure the flow or quantity. For illustration, suppose you have a tank of water on the roof of a house, with an outlet down near the ground. When the water is flowing, the amount of water that comes out of the stopcock would represent the ampères, and the pressure of the water in the tank would represent the voltage of your current. Whatever resistance there might be to the flow of water would be represented by ohms.

*Cataphoric Medication.*—In speaking of cataphoric medication no attempt will be made to touch upon its relations to general medicine and surgery, as time will not permit. The transactions of the American Electro-Therapeutic Association for the past few years, Bigelow's book, to which I have already referred, and the recently published articles on this general subject in the various medical and dental journals, will give you sufficient information along this line.

Cataphoric medication is of great interest to you in its relations to dentistry, and one of its most important applications is in the obtunding of sensitive dentine. In this word "obtunding" you have a term that is most appropriate. No term used by physicians really expresses the idea so well as this word obtunding. There are several uses to which cataphoresis has been applied in dentistry, but to my mind the most important application of it is the obtunding of sensitive dentine. It would be foolish for me in this presence to undertake to go into the details of obtunding sensitive dentine. You will find them set forth in Dr. Gillett's articles already referred to, and which you have all doubtless read. I have witnessed the application of the electric current with cocaine for this purpose, and in many cases where it was very successful, and you have in this agent a means of doing your work with the least possible discomfort to the people who come under your hands. I do not believe you dentists fully realize the extent of the suffering and the nervous dread that prevails among your patients. If you could fully realize the nervous tension to which they are subjected and the dread of the operations they undergo in the dental chair you would not hesitate to adopt any means which gives hope of relief from pain, and it would seem before many months your patients will begin to demand that your operations be done by this method.

Another application of it has been made in connection with crown- and bridge-work. It has also been applied in several instances to obviate the pain in the extraction of teeth, and very favorable reports of its use in such cases have been given. In the obtunding of sensitive dentine we have the best and most practical illustration of the actual application of cataphoresis that mankind has yet made use of. Heretofore dentists have really had nothing that they could honestly say would obtund sensitive dentine. Now by the application of the continuous current cocaine can be introduced and made to do this successfully. So far as I have been able to make out, the practical application of the continuous current in the obtunding of sensitive dentine and the bringing of this question before the dental profession in such a way that the profession can understand it and make use of it is due to Dr. Henry W. Gillett, of Newport, and the credit belongs to him.

I have made some use of cataphoresis in the line of my special work in the nose, throat, and ear. At the Manhattan Eye and Ear Hospital I have a case of suppurating antrum of Highmore in which a test was made. This patient gave a peculiar history. You know that Professor Garretson held that most, if not all, cases of antrum disease are due to caries of the teeth. I have seen so many cases of suppuration of the antrum where the teeth gave every evidence of being perfectly sound that I am fully convinced that a considerable proportion of these cases have nothing to do with the teeth. This case was a man who was a watchmaker by trade, and every day at about ten o'clock he had a profuse discharge of pus from the right nostril. That was about the only symptom.

In his occupation of watchmaker he began to work about nine o'clock in the morning, with his head over the bench, in which position the normal opening of the antrum, the ostium maxillare, would be in the dependent portion, and after he was working an hour or so pus would begin to flow out. Percussion over the region of the antrum on both sides revealed some tenderness on the right side. He was then taken into a dark room[1] and transilluminated with this little electric lamp, and found the reflected light much dimmer on the affected side underneath the eye, and from these indications I made up my mind that there was suppuration of the antrum. A cataphoric application of a ten-per-cent. solution of cocaine was made to the canine fossa, using the Wheeler fractional volt-selector. After fifteen minutes, during which time the current was gradually increased to fifteen volts, an opening was made into the antrum without pain to the patient.

It is hardly necessary for me to say that after the opening was made there was a profuse discharge of pus. You can also use the continuous current in this manner for the obtunding of soft tissues.

For extraction there should be used a double positive electrode upon each side of the tooth, and the negative electrode upon some other portion of the body.

Cataphoresis has been used in the treatment of pyorrhœa alveolaris and other suppurative diseases, necrosis, and various kinds of ulcers. You can use it with various remedies to destroy germ-life, also for sterilizing and bleaching, and there are new applications of it yet to come. Enough has been done to show that there is great utility in this application of electricity, and I do not overstate the probabilities in saying that in sustaining the reputation which this Society has acquired as an energetic body of investigators some member will find still new applications and accomplish new things with this agent.

Now, to proceed with cataphoric medication. Attention is called to the different kinds of remedies that may be made use of. First and foremost among all the medicines must stand that remedy which has become so helpful to dentists and physicians in the obtunding of the soft tissues,—cocaine,—a remedy without which I should want to give up the specialty which I practise. Fresh solutions of pure cocaine are indispensable. Some manufacturers do not seem to be able to eliminate the irritating qualities. You will find it a good plan to purchase your cocaine in crystals and make the solution just at the time of using.

In the use of cocaine in dentistry most of you know the way to apply it. Use a strong solution, placing in the cavity to be ex-

---

[1] See Dental Cosmos, January, 1891.

cavated a pledget of cotton saturated with it, and then apply the current. Dr. Gillett recommends a twenty-per-cent. solution, or clear crystals, with just enough water added to form a solution; then apply the positive electrode, and turn on the current.

You will wonder why it is that you can increase the current from time to time without great discomfort to the patient. The reason is that the near areas of tissue become obtunded, and are consequently able to bear a stronger current than at first. Then you turn on the current and carry the obtunding agent into more distant portions. It has been objected that the time required to obtund a sensitive tooth is considerable; but Dr. Gillett assured me in conversation that he had found the time saved in excavating would overbalance the time lost in obtunding.

Morton recommends guaia-cocaine as being more rapidly diffusible under the pressure of the current. In its present form it cannot be used upon mucous surfaces on account of its escharotic effects, but this would not obtain in the dentine. The odor is most objectionable,—an objection, however, which may be overcome by combining with the oil of pine.

While on this subject a few words upon the toxic effects of cocaine may not be out of place, and also of its non-action in some cases. The toxic effect you are liable to get if you use it on mucous surfaces. During the history of its use in the Manhattan Eye and Ear Hospital in many operations it has not been uncommon to get its toxic effects. This is not serious, as a rule. With a properly-constructed rubber dam, and the use of the cocaine in the dentine alone, where it is somewhat difficult to get enough into the tissue to obtund it, it is quite impossible to get sufficient cocaine into the general circulation to produce any toxic effect.

Another thing that you must take into consideration is the fact that in some cases cocaine seems to have no effect of any kind. In your work you will sometimes meet such patients who will present this physiological condition; therefore if you occasionally meet with a case where cataphoric obtunding fails you will bear this fact in mind. When you intend to resort to cataphoric medication and use other remedies besides cocaine, it would be a wise precaution to obtund the tissue with a cataphoric application of cocaine before using the other remedies; you can carry up the current much stronger in that way than otherwise, and the stronger the current the greater the obtunding effect.

I might speak of the various properties of hydrogen peroxide, $H_2O_2$, which has been much used for bleaching purposes. As far as my reading goes, there are no preparations but these—hydrogen peroxide and pyrozone—with which you can do the work known as bleaching, which is only accomplished by cataphoresis. These remedies are also useful as germicides.

In this connection attention is called to a remedy which has proved so helpful to me in nose-, throat-, and ear-diseases that I feel sure that there is a field for it in dental practice, especially those conditions attended with more or less septic developments, such as pyorrhœa alveolaris, caries, aphthous-ulcers, etc. I refer to borolyptol, manufactured by the Palisade Manufacturing Company, a *well-known, non-toxic, non-irritant destroyer of micro-organisms and spores.* The tests of two bacteriologists would indicate that its germicidal powers equal and in some instances exceed that of a 1 to 1000 solution of corrosive sublimate.

During the past few days I have made use of it, by means of cataphoresis, in three cases which may interest you. One was an Italian, about fifty years old, with badly neglected teeth, several of which were decayed, black in color, and two or three old roots were resting loosely in the gums. He had recently had a rheumatic attack, accompanied by a severe tonsillitis. The gums were retracted and boggy, and he complained of some pain around the teeth. For experimental purposes I used borolyptol cataphorically, first anæsthetizing the surface of the gums with a short cataphoric application of cocaine. The borolyptol was applied full strength to the entire surface of the gums, and wherever there were any appearances of disease. The Wheeler fractional volt-selector was employed, and this patient could stand a rapid increase of current up to twenty-five volts. The cotton pledget was frequently exchanged to allow of fresh borolyptol. The odor and soreness were much relieved. I have also experimented with two other cases furnished to me by Dr. H. W. F. Cady, of New York.

The first case was a child two years of age, who had suffered with a severe attack of measles six weeks previously. She was a badly-nourished, scrofulous-looking child, and three weeks after the measles she had a necrosis of the middle portion of the superior maxillary bone. Dr. Cady removed two teeth and a portion of necrosed alveolar process.

There was still quite a large surface of denuded bone, with tendency to sloughing of soft tissues. The odor was very bad and the adjacent teeth covered with sordes. Applied borolyptol diluted one-half, after cocaine, using the volt-selector, and carrying the voltage up to sixteen. The cotton and solution were frequently changed, and the application was not only made to the ulcerating surface but to the entire gum surface, the whole procedure occupying fifteen minutes. Two days later the patient returned for another treatment, and the mother said the child had suffered much less pain, had been less fretful, had eaten and slept better, and that there was no odor for twenty-four hours after treatment, but it had partially returned. Amount of denuded surface about the same, but the teeth were much cleaner. The treatment was repeated, using this time full strength borolyptol. Returned two days later, with improvement, as far as condition of wound, general physical condition, and odor are concerned. Bone is still denuded.

The last case was one of aphthous sore mouth and necrosis of large sections of both superior and inferior maxillary bones, also following an attack of measles two months previously. Dr. Cady had already removed a large section of necrosed superior maxillary bone, and kindly deferred further treatment in order to allow me to experiment with borolyptol applied by cataphoresis.

There was a large aphthous ulcer on the inner surface of the lower lip, very dark and foul, necrosis of inferior maxillary bone, gums boggy and hemorrhagic, teeth covered with sordes, odor very bad. Child had high fever, and submaxillary glands swollen and very painful. Made a cataphoric application with cocaine, low voltage over entire surface for two minutes, then borolyptol diluted one-half, increasing current gradually to fourteen volts, after which the surfaces presented a much cleaner appearance. Two days later the mother says child seemed relieved after former treatment, there was less odor, slept all the afternoon, and ate more than usual; but twenty-four hours afterwards symptoms had all returned, and to-day the mouth appears about the same as at former treatment, except the ulcer is not quite so large and not so unhealthy in appearance and the teeth are cleaner. Repeated treatment, using borolyptol full strength, and increasing current to sixteen and a half volts, very thoroughly and for about twenty minutes. Three days later reports that there has been less pain in mouth, but the submaxillary glands became very painful, and Dr. Cady removed a tooth and piece of necrosed process, after which the glands were better. The ulcer was rapidly healing, and the general condition of gums much improved. I feel sure that cataphoric medication in cases similar to the ones described, using borolyptol, forms a useful adjunct to the necessary treatment by operative interference, and also hastens the separation of the dead bone.

*Question.*—Did you apply the borolyptol cataphorically?

*Dr. Phillips.*—Yes.

Dr. Gillett's first experiments and demonstrations were made with an ordinary adapter. In showing him the apparatus which I had, and which was used for the ordinary purposes of the continuous current, he said he thought that it might be used in obtunding sensitive dentine. The next day he brought a patient, the first experiment was made, and it was successful. But we found that when the current was increased the patient experienced pain, and he thought it was necessary to overcome those jumps of the current in order to make a perfect application of cataphoresis. A successful application of cataphoresis in obtunding must depend upon the relief of one pain without the introduction of another. Dr. Gillett purchased one of the same adapters. He made some changes in it from time to time, but finally found it would not answer the purpose unless the current could be so controlled that the changes in its gradations or steps would not be painful to the patient. He called to his assistance Mr. G. M. Wheeler, of the Electro-Therapeutic Company, and explained what he wanted. The result of his suggestion, in conjunction with Mr. Wheeler's mechanical and electrical skill, is the instrument known as the Wheeler fractional volt-selector. You are constantly hearing the words "rheostat," "adapter," and "cell-selector."

A rheostat is an adjustable resistance introduced into the circuit in simple connection with the patient to control the quantity of current flowing, without regard to steady voltage. An adapter is an elaborated rheostat, for use with the various street currents. A cell-selector is a series of contact points over which travels a sliding switch-arm, each point connecting with one or more cells of a series battery. These cells ordinarily represent about two volts of pressure each.

The difference between an adapter or rheostat and a fractional volt-selector can best be explained by means of a simple illustration like the following:

Imagine *a* to be a dam of water and *b* the sliding gate. Now, suppose you raise the sliding gate one step from the bottom and a current of water will flow; but the opening being at the bottom of the volume of water in the dam, there will be behind it the pressure of the weight of the volume of water above. Raise the gate another step, and there will be an increased flow of water with practically the same pressure.

On the other hand, if the gate should be lowered from the top one step, there would be a flow of water, but the pressure would be diminished by the difference in weight of water at the upper and lower openings of the gate. Now, in electricity, the raising of the gate from the bottom would represent the adapter or rheostat, on account of the entire pressure being back of it all the time. The lowering of the gate from the top represents the fractional volt-selector, because there is a gradual increase in the pressure as you lower the gate.

The fractional volt-selector differs from the adapter or rheostat as the force of water would differ if instead of raising your gate from the bottom you were to let it down from the top when you want a flow of water. If you let it down one step you have a flow of water with a low pressure. And if you let it down another step you have another flow of water with a little greater pressure, and so on. By means of the fractional volt-selector you can let on the pressure of the current by small gradations, beginning with no pressure and adding a little more pressure, and a little more, as you require. I have not seen anything that has been so effective in controlling the current as this apparatus.

There are numerous cell-selectors on the market. With them you can make use of any number of cells desired,—one volt and a half or two volts to each cell,—turning on another cell as you want an increased current. This will give jumps of pressure of one and a half to two volts, corresponding to the type of battery in use, resulting in painful shocks to the patient. These will not work well for obtunding purposes.

In the selection of such instruments one needs to be very careful. And for this particular kind of work, unless the instrument will permit the increase of the current in fractions of a volt, you cannot successfully use it. Dr. Gillett has told me that he has found patients to whom even half-volt steps would be very painful, and it is those delicate and sensitive patients who especially need this release from pain. Dr. Gillett says the voltage must be turned on in steps of less than half a volt. With a cell-selector you turn on one and a half or two volts at a time, and that does not answer the purpose.

Volt-selector.

The fractional volt-selector will give you gradations of less than one-quarter of a volt, and with that perfect control of the current the possibility of giving pain to the patient is extremely remote. After careful experiments I have found that it will do the work.

This fractional volt-selector is manufactured by the Electro-Therapeutic Company, of New York, and is made to use with the street current of one hundred and ten volts. It is so arranged that there is no danger of shock from a possible crossing of wires. For those who are not able to make use of the street current the manufacturers furnish a box of cells, which will give forty volts, and can be used in the same way as the street current.

In using the Edison current you may have a little trouble to know which is the positive pole. If you use the battery cells this trouble is obviated, the positive pole being definitely fixed and marked. To determine the positive pole, take a strip of blotting-paper, soaked with a solution of iodide of potassium, and press it down on the two poles of the circuit to be tested, and leave it for a moment, and when it is taken away one end of the paper will be dark. The dark end indicates the positive pole. It means that there is a liberation of iodine from the iodide of potassium by the action of the current.

Morton's straight applicator.

Morton's tong-shaped duplex.

Morton's mouth cataphoric electrode.

Dr. Younger,[1] of San Francisco, has performed the operation of implantation successfully in connection with cataphoresis. He had been in the habit of using hypodermic injections of cocaine before performing the operation of implantation, but in two cases he used the cocaine cataphorically, using the fractional volt-selector in making the application, and the operation was performed with much more satisfactory results, and he told me he had no doubt that in the future this appliance would be used in such operations.

---

[1] Dental Cosmos, January, 1896, page 50.

In the operation of extracting teeth the obtundent will also have to be applied to the gum in order to obtund the entire area.

The following is the platinum point and handle as used by Dr. Gillett for obtunding purposes.

Here is an electrode which seems to have a certain bend to suit the dentist. This one was made upon lines suggested by Dr. William Carr, of New York.

## Figures and Tables

**Figure f1:**
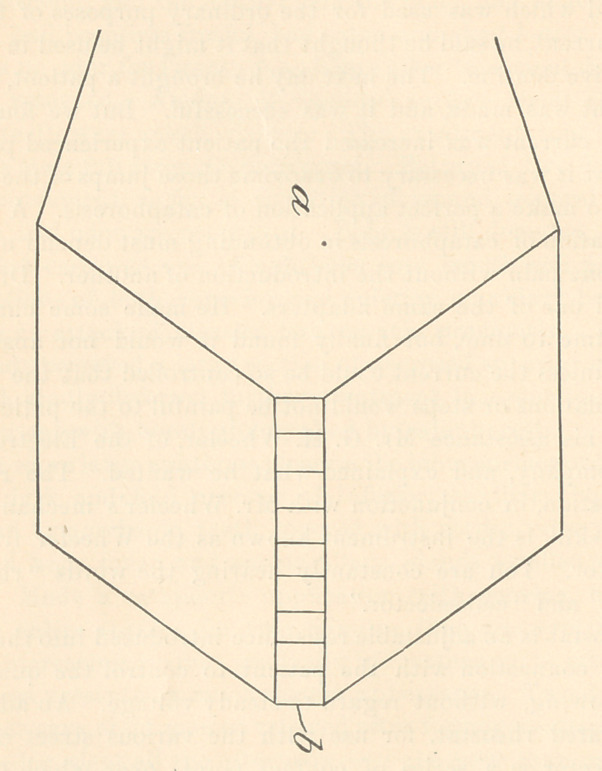


**Figure f2:**
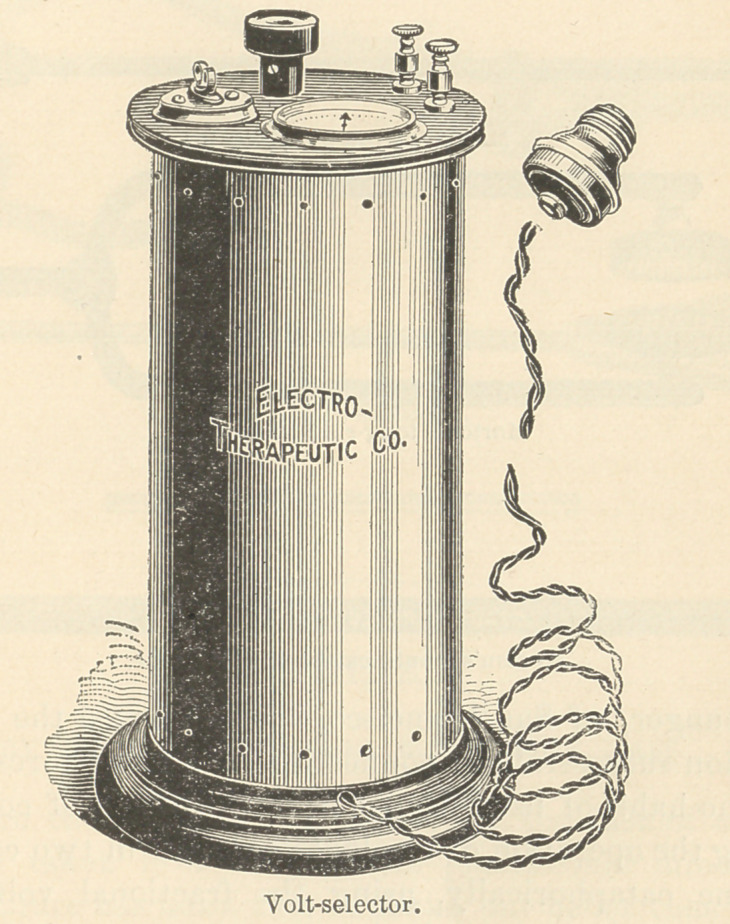


**Figure f3:**
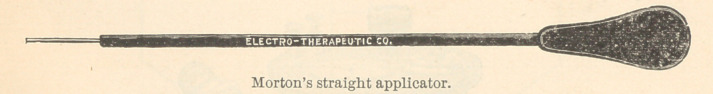


**Figure f4:**
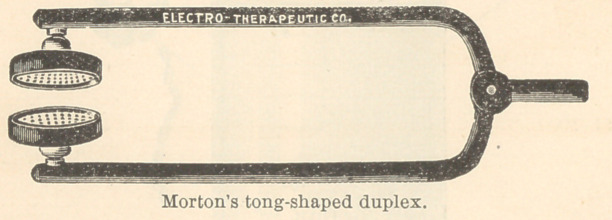


**Figure f5:**
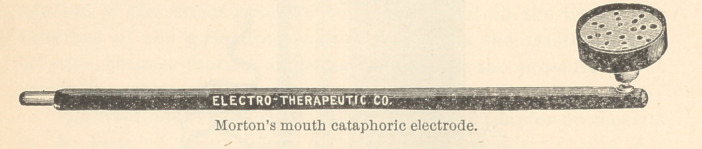


**Figure f6:**
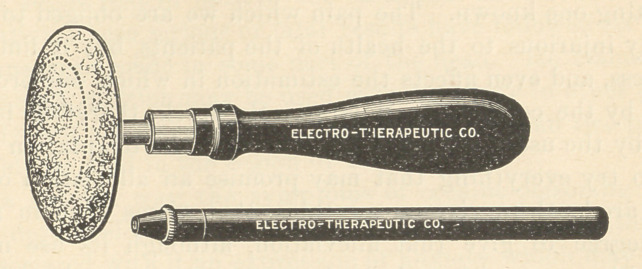


**Figure f7:**